# Continuous Flow Bioamination of Ketones in Organic Solvents at Controlled Water Activity using Immobilized ω‐Transaminases

**DOI:** 10.1002/adsc.201901274

**Published:** 2020-02-17

**Authors:** Wesley Böhmer, Alexey Volkov, Karim Engelmark Cassimjee, Francesco G. Mutti

**Affiliations:** ^1^ Van't Hoff Institute for Molecular Sciences, HIMS-Biocat University of Amsterdam Science Park 904 1098 XH Amsterdam The Netherlands; ^2^ EnginZyme AB Tomtebodavägen 6 171 65 Solna Sweden

**Keywords:** asymmetric synthesis, biocatalysis, α-chiral amines, flow chemistry, transaminases

## Abstract

Compared with biocatalysis in aqueous media, the use of enzymes in neat organic solvents enables increased solubility of hydrophobic substrates and can lead to more favorable thermodynamic equilibria, avoidance of possible hydrolytic side reactions and easier product recovery. ω‐Transaminases from *Arthrobacter sp*. (AsR−ωTA) and *Chromobacterium violaceum* (Cv−ωTA) were immobilized on controlled porosity glass metal‐ion affinity beads (EziG) and applied in neat organic solvents for the amination of 1‐phenoxypropan‐2‐one with 2‐propylamine. The reaction system was investigated in terms of type of carrier material, organic solvents and reaction temperature. Optimal conditions were found with more hydrophobic carrier materials and toluene as reaction solvent. The system's water activity (a_w_) was controlled via salt hydrate pairs during both the biocatalyst immobilization step and the progress of the reaction in different non‐polar solvents. Notably, the two immobilized ωTAs displayed different optimal values of a_w_, namely 0.7 for EziG^3^−AsR−ωTA and 0.2 for EziG^3^−Cv−ωTA. In general, high catalytic activity was observed in various organic solvents even when a high substrate concentration (450–550 mM) and only one equivalent of 2‐propylamine were applied. Under batch conditions, a chemical turnover (TTN) above 13000 was obtained over four subsequent reaction cycles with the same batch of EziG‐immobilized ωTA. Finally, the applicability of the immobilized biocatalyst in neat organic solvents was further demonstrated in a continuous flow packed‐bed reactor. The flow reactor showed excellent performance without observable loss of enzymatic catalytic activity over several days of operation. In general, ca. 70% conversion was obtained in 72 hours using a 1.82 mL flow reactor and toluene as flow solvent, thus affording a space‐time yield of 1.99 g L^−1^ h^−1^. Conversion reached above 90% when the reaction was run up to 120 hours.

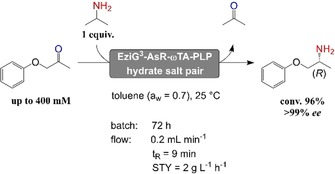

## Introduction

The use of enzymes in laboratory and industrial scale organic synthesis has predominantly been restricted to either monophasic aqueous reaction media in presence of an optional water‐miscible cosolvent or bi‐phasic aqueous−organic media or reverse‐micelles systems in which the enzyme is still dissolved in a significant amount of water.^1^ However, many different enzyme families have been applied in neat organic solvents or supercritical gases over the last few decades,^2^ whereas more recent contributions entail the use of ionic liquids^3^ and deep eutectic solvents.^4^ Notably, the use of enzymes in non‐aqueous media enables the avoidance of some drawbacks of biotransformations in an aqueous buffer, such as the low solubility of hydrophobic substrates and the possible occurrence of unwanted hydrolytic side reactions; in certain cases, thermodynamic equilibria can become more favorable in a non‐aqueous medium and product recovery can also be facilitated.^5^ In this context, enzyme immobilization and microenvironment optimization have proven to enhance stability and catalytic performance in neat organic solvents.^6^ However, it is important to remark that dynamic and catalytic properties of enzymes in non‐aqueous media are dependent on the presence of a limited but critical amount of water.^7^ On the one hand, less than a monolayer of water is required for an isolated enzyme molecule to show activity in an organic solvent. On the other hand, the inherent conformational rigidity of an enzyme in an organic medium with minimal water content preserves its native folding state.^8^ However, the impact of these factors significantly depends on the biocatalyst form, which can be either an isolated enzyme as lyophilized powder, or immobilized on a carrier material, or entrapped in a polymeric matrix, or even a whole cell biocatalyst.

Hydrolases were the first and remain the most frequently applied enzymes in neat non‐aqueous environments.2b, 9 Oxidoreductases, including alcohol dehydrogenases^10^ and peroxidases^11^ as well as alcohol and phenol oxidases^12^ were found to be active in neat organic solvents in the 1980s, and further studies on oxidoreductases were conducted during this decade.^13^ Examples with lyases comprise hydroxynitrile lyases,^14^ cyclases,^15^ decarboxylases,13d, 13e, 16 hydroperoxide lyases^17^ and benzaldehyde lyases.^18^

In contrast, transferases such as ω‐transaminases (ωTAs) have only sporadically been applied in neat organic solvents. Transaminases are pyridoxal phosphate‐dependent (PLP) enzymes that enable the transfer of an amino group from a simple donor molecule to a prochiral carbonyl compound, thereby generating a wide range of high‐value chiral α‐amine containing compounds.^19^ Transaminases possess significant potential for industrial application due to high turnover rates, excellent enantioselectivity and economical cofactor (PLP) regeneration.^20^ An engineered and immobilized ωTA was applied for the production of Sitagliptin in a neat organic solvent.^21^ In a concomitant publication, we reported that wild‐type ωTAs retain elevated catalytic activity for the amination of prochiral ketones in neat organic solvents when applied as lyophilized cell extracts.^22^ The activity of the ωTA's lyophilized cell extract was retained by the addition of a small amount of water below the saturation value in an organic medium. The system exhibited a more favorable thermodynamic equilibrium for the amination compared to the same reaction in aqueous buffer as well as an apparent lack of substrate inhibition, and it enabled simple product recovery along with biocatalyst recycling. In later studies, the same strategy was exploited for the synthesis of enantiomerically pure 3‐substituted cyclohexylamines and both enantiomers of valinol.^23^ *E. coli* whole cells expressing ωTAs were then applied in a flow reactor.^24^ Finally, ωTAs have recently been implemented in neat MTBE as enzyme particles coated with ionic liquid.^25^

To the best of our knowledge, there are no reports about the application of immobilized ωTAs (as isolated enzymes) in neat organic solvents and continuous flow reactors. In a previous study, we optimized the immobilization of transaminases on controlled porosity glass carrier material (EziG) for their application in an aqueous environment.^26^ Herein, we report the application of both an (*R*)‐selective and an (*S*)‐selective transaminase as immobilized enzymes in neat organic solvents at controlled a_w_ (water activity). In fact, the residual amount of water in a given solvent (i. e., below the water saturation level) is critical in non‐aqueous biocatalysis, and this water partitions between all components in the system, including the solvent, enzyme, support matrix and the headspace of the reaction vessel. The water activity (a_w_) is a more reliable parameter than the water content (c_w_) for analysis and comparison of enzyme performance in different solvents because the hydration state of an enzyme is always fixed by the a_w_ value independently of the solvent of choice.^27^ Optimization of the reaction system was performed in terms of carrier material, solvent and reaction temperature. Under optimal reaction conditions, the immobilized (*R*)‐selective transaminase was employed in a packed‐bed flow reactor in a neat organic solvent showing high catalytic performance.

## Results and Discussion

Two stereocomplementary ωTAs, namely the (*R*)‐selective ωTA from *Arthrobacter sp*. (AsR−ωTA)^28^ and the (*S*)‐selective ωTA from *Chromobacterium violaceum* (Cv−ωTA)^29^ were chosen for application in neat organic media. In this study, we decided to use purified ωTAs in order to be able to quantify the amount of immobilized enzyme loaded on the carrier material with high precision and accuracy. However, the efficiency of the selective immobilization of ωTA from crude lysate on EziG^3^ has already been demonstrated.^26^ Therefore, we applied a typical enzyme loading of 10% w w^−1^ ωTA. The immobilization of ωTAs was performed on three types of EziG polymer‐coated controlled porosity glass carrier materials possessing distinct surface properties. The first type of material, henceforth called EziG^1^ (Fe Opal), is covered with a hydrophilic polymeric surface. EziG^2^ (Fe Coral) has a hydrophobic surface polymer and EziG^3^ (Fe Amber) is covered with a semi‐hydrophobic polymer surface (Table S3). The immobilization of ωTAs was performed by incubating the desired amount of enzyme in buffer supplied with EziG carrier material according to our previously reported protocol.^26^ The progress of the immobilization was determined by measuring the amount of enzyme remaining in the immobilization buffer with a Bradford assay (see SI – section 2.2). Quantitative immobilization of AsR−ωTA on EziG carrier materials was obtained within three hours of incubation.^26^

A practical method for a_w_ control involves the use of insoluble salts possessing a varied stoichiometry of co‐crystallized water molecules. By incubating the organic reaction medium with a specific pair of salts, it is possible to dynamically “buffer” the water content during the reaction and thus fix the a_w_. A selection of salt hydrate pairs for setting a_w_ is available from the literature.^30^

Hydrophilic carrier materials tend to retain a significant amount of water. Therefore, in the present study, the water content (c_w_) of EziG‐immobilized ωTA had to be lowered after the immobilization step and controlled during the reaction in organic solvent in order to obtain catalytic activity. Our previously reported methodology based on the lyophilization of ωTAs as cell extracts was not applicable for EziG‐immobilized enzymes and resulted in the complete loss of catalytic activity.^22^ Other attempts aimed at reducing the water content of the immobilized ωTA by the gentle flowing of a stream of air or dinitrogen were also unsuccessful. Optimizing c_w_ (and consequently a_w_) in the immobilized ωTA using salt hydrate pairs proved to be successful, particularly in combination with a hydrophilic solvent. The reason for using a hydrophilic solvent is to gently and gradually remove the excess of water from the enzyme‐carrier complex while preserving enzyme activity. Optimal c_w_ could be set in the system via simple washing of the wet immobilized ωTA with hydrophilic organic solvent in the presence of salt hydrate pairs (i. e., Na_2_HPO_4_ ⋅ 2H_2_O/Na_2_HPO_4_ ⋅ 7H_2_O or Na_2_HPO_4_ ⋅ 7H_2_O/Na_2_HPO_4_ ⋅ 12H_2_O). Then, the hydrophilic solvent was removed and hydrophobic solvents (also pre‐equilibrated at the set a_w_ using the same type of salt hydrate pairs) were instead applied during the reaction to prevent the further stripping of enzyme‐bound water, thereby ensuring high catalytic activity of the immobilized ωTA.

**Scheme 1:**
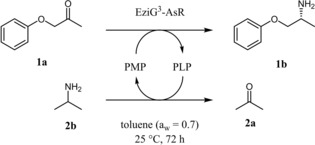
Reductive amination of 1‐phenoxypropan‐2‐one (**1 a**) with 2‐propylamine (**2 b**) catalyzed by EziG^3^‐AsR−ωTA in neat organic solvents at controlled a_w_.

The transamination of 1‐phenoxypropan‐2‐one (**1 a**, 50 mM) using 2‐propylamine **2 b** (150 mM) as the amine donor was used as a model reaction (Scheme 1). In particular, the amination of these types of substrates is thermodynamically favored in non‐aqueous environment as demonstrated in the previous study that was conducted using lyophilized crude enzyme in batch system.^22^ Among the others, the amine product **1 b** is an analogue of the drug Mexiletine.29b

Application of a lyophilized crude cell extract of AsR−ωTA in MTBE was shown to best operate at a water activity of a_w_=0.6.^22^ However, setting the hydration state of immobilized EziG^3^−AsR−ωTA with MTBE (a_w_=0.6), followed by transamination either in toluene (a_w_=0.6) or MTBE (a_w_=0.6) resulted in only 4% and 8% conversions, respectively (Table 1, entries 1–2). A significant increase in conversion (28%) was observed when EtOAc (a_w_=0.6) was used to set the hydration state of EziG^3^−AsR−ωTA and the immobilized enzyme was then applied in toluene (a_w_=0.6) (Table 1, entry 3). Although immobilized EziG^3^−AsR−ωTA was obtained in a sufficiently active form using EtOAc at a_w_ of 0.6 for equilibration, we postulated that some additional water molecules could be adsorbed by the immobilized ωTA over time, thus reducing its catalytic activity during the progress of the reaction. Indeed, when a pair of disodium hydrogen phosphate di‐ and hepta‐hydrate salts was added to the reaction mixture (1:1, w w^−1^ ratio salt hydrate pair to immobilized ωTA), we observed a remarkable increase in conversion (87%, Table 1, entry 4). The addition of the salt hydrate pairs to the reaction mixture also significantly increased the system's reproducibility; therefore, all further experiments were performed with salt hydrate pairs in the reaction.

**Table 1:** Optimization of conditions for the transamination of **1 a** (50 mM) with **2 b** (150 mM) catalyzed by EziG^3^−AsR−ωTA (22 mg, enzyme loading: 10% w w^−1^) in neat organic solvents (1 mL) at controlled water activity (a_w_) as specified, at 25 °C and shaking on an orbital shaker at 900 rpm for 72 h. 900 rpm agitation was selected because the immobilized enzyme appeared to have the best contact with the solvent at this rate (i. e., all the beads were evenly distributed in the heterogeneous system and not prevalently at the bottom of the reaction mixture).

Entry	Immobilization buffer	a_w_ equilibration solvent	Reaction solvent	Salt hydrate pairs	Conv. [%]^3^
1	KPi (100 mM, pH 8.0)	MTBE^1^	MTBE^1^	none	4±0
2	KPi (100 mM, pH 8.0)	MTBE^1^	Toluene^1^	none	8±1
3	KPi (100 mM, pH 8.0)	EtOAc^1^	Toluene^1^	none	28
4	KPi (100 mM, pH 8.0)	EtOAc^1^	Toluene^1^	Na_2_HPO_4_ ⋅ 2H_2_O/Na_2_HPO_4_ ⋅ 7H_2_O	87
5	KPi (100 mM, pH 6.0)	EtOAc^2^	Toluene^2^	Na_2_HPO_3_ ⋅ 5H_2_O/Na_2_HPO_4_ ⋅ 7H_2_O	88±6
6	KPi (100 mM, pH 6.5)	EtOAc^2^	Toluene^2^	Na_2_HPO_3_ ⋅ 5H_2_O/Na_2_HPO_4_ ⋅ 7H_2_O	81±14
7	KPi (100 mM, pH 7.0)	EtOAc^2^	Toluene^2^	Na_2_HPO_3_ ⋅ 5H_2_O/Na_2_HPO_4_ ⋅ 7H_2_O	92±4
8	KPi (100 mM, pH 7.5)	EtOAc^2^	Toluene^2^	Na_2_HPO_3_ ⋅ 5H_2_O/Na_2_HPO_4_ ⋅ 7H_2_O	85±7
9	KPi (100 mM, pH 8.0)	EtOAc^2^	Toluene^2^	Na_2_HPO_3_ ⋅ 5H_2_O/Na_2_HPO_4_ ⋅ 7H_2_O	96±1
10	MOPS (100 mM, pH 7.5)	EtOAc^1^	Toluene^1^	Na_2_HPO_4_ ⋅ 2H_2_O/Na_2_HPO_4_ ⋅ 7H_2_O	21±2
11	HEPES (100 mM, pH 7.5)	EtOAc^2^	Toluene^2^	Na_2_HPO_3_ ⋅ 5H_2_O/Na_2_HPO_4_ ⋅ 7H_2_O	75±5
12	Tris (100 mM, pH 7.5)	EtOAc^2^	Toluene^2^	Na_2_HPO_3_ ⋅ 5H_2_O/Na_2_HPO_4_ ⋅ 7H_2_O	91±2

1 a_w_=0.6. 2 a_w_=0.7. 3 Conversion to amine product in 72 h reaction time. Values are depicted with standard deviations over three independent experiments. All reactions afforded >99% enantiomeric excess of the amine product.

Enzyme ionization influences the catalytic activity of enzymes in non‐aqueous media.2b In particular, the effect of the pH memory of the lyophilized enzymes was studied by tuning the ionization state of their functional groups in aqueous buffers prior to lyophilization and submerging them in a non‐aqueous medium.^31^ Although the pH of the immobilization buffer did not significantly affect the catalytic activity of EziG^3^−AsR−ωTA immobilized from KPi buffers (Table 1, entries 5–9), we observed a significant drop in conversion when MOPS buffer was used for the immobilization (21%, Table 1, entry 10). The use of KPi buffer (100 mM, pH 8.0) led to a remarkable 96% conversion; therefore, further studies were performed using these conditions. Finally, EziG^3^−AsR−ωTA immobilized from HEPES buffer (100 mM, pH 7.5) and Tris buffer (100 mM, pH 7.5) resulted in 75% and 91% conversions, respectively (Table 1, entries 11–12).

**Figure 1:**
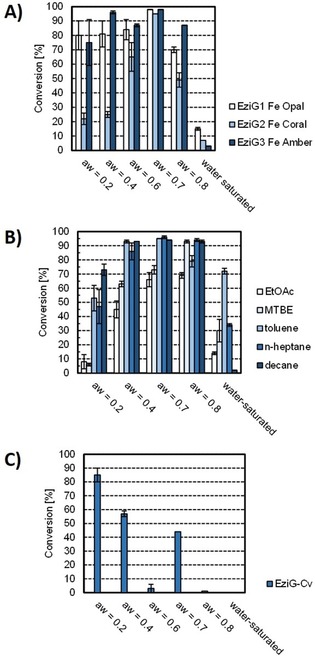
Performance of immobilized ωTA in neat organic solvents at controlled and varied a_w_ values. **A)** AsR−ωTA on three EziG carrier materials in toluene, **B)** EziG^3^−AsR−ωTA applied in different reaction solvents and **C)** EziG^3^−Cv−ωTA applied in toluene. Immobilization conditions: EziG (as specified, 20 mg), AsR−ωTA (2 mg, 54 nmol) or Cv−ωTA (2 mg, 38 nmol); enzyme loading: 10% w w^−1^, KPi buffer (1 mL, 100 mM, pH 8.0), PLP (0.1 mM), 4 °C, 120 rpm; incubation time: 3 h. Reaction conditions: EziG^3^−AsR−ωTA or EziG^3^−Cv−ωTA (22 mg, enzyme loading: 10% w w^−1^), hydrate salts (ca. 25 mg), reaction solvent (1 mL, a_w_ as specified), **1 a** (50 mM), **2 b** (150 mM), 25 °C, 900 rpm; reaction time: 72 h. Values are depicted with standard deviations over three experiments. All reactions afforded >99% enantiomeric excess of the amine product.

Most immobilization supports absorb water and the use of different support materials can have a direct effect on the catalytic activity and stability of enzymes in non‐aqueous media. For example, subtilisin Carlsberg covalently attached to macroporous acrylic supports was demonstrated to exhibit improved catalytic activity at high c_w_ as a result of water absorption by the support material.^32^ EziG‐immobilized ωTAs (EziG^1^−AsR, EziG^2^−AsR, and EziG^3^−AsR, Table S3) were applied in toluene at a_w_ controlled by salt hydrate pairs (Table S4); no hydrate salts were added when water‐saturated solvents were used. At comparable levels of a_w_, the use of EziG^1^−AsR−ωTA and EziG^3^−AsR−ωTA resulted in higher conversions compared with EziG^2^−AsR−ωTA (Figure 1A and Table S5). However, a general trend was observed whereby all three EziG carrier materials showed higher conversions at increased a_w_, with an optimal performance at a value of 0.7 and drastically lower conversions in water‐saturated toluene. Notably, more hydrophilic EziG^1^−AsR−ωTA also performed well at low a_w_, thereby supporting the fact that water adsorption by the carrier material enhances the immobilized ωTA's performance in solvents with low water content. Conversely, hydrophobic EziG^2^−AsR−ωTA exhibited a dramatic drop in conversion at low a_w_. EziG^3^ (Fe Amber) was chosen as the carrier material for further studies due to its superior performance in the range of 0.4≤a_w_≤0.7. Notably, the a_w_ values that are reported in this work with one significant digit must be considered as accurate because the original literature values are reported with an error on the second significant digit.^30^

**Figure 2:**
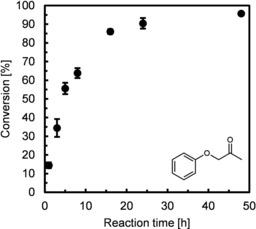
Time study of EziG^3^−AsR−ωTA for the transamination of **1 a** in toluene at controlled a_w_ of 0.7. Immobilization conditions: EziG^3^ (Fe Amber, 20 mg), AsR−ωTA (2 mg, 54 nmol; enzyme loading: 10% w w^−1^), KPi buffer (1 mL, 100 mM, pH 8.0), 4 °C, 120 rpm; incubation time: 3 h. Reaction conditions: EziG^3^−AsR−ωTA (22 mg, enzyme loading: 10% w w^−1^), salt hydrate pairs (ca. 25 mg), reaction solvent (1 mL, a_w_=0.7), **2 b** (150 mM), **1 a** (50 mM), 25 °C, 900 rpm; reaction time: 72 h. All reactions afforded >99% enantiomeric excess of the amine product.

Another important parameter that determines the hydration state of immobilized enzymes in non‐aqueous media is the solvation of water molecules by the reaction solvent. In general, hydrophobic solvents (i. e., solvents with a high log P value) serve as better candidates because they lack the ability to strip the enzyme of essential water molecules.31b, 33 EziG^3^−AsR−ωTA was tested in different organic solvents at controlled a_w_ in order to define the optimal solvent for our system (Figure 1B). Organic solvents were chosen with log P values ranging from 0.7 to 5.6 (Table S6). EtOAc and MTBE with log P values of 0.7 and 0.9 respectively showed moderate to good conversions, whereas *n*‐heptane and *n‐*decane (log P of 4.0 and 5.6 resp.) performed significantly better. A recent assessment of the “greenness” of these solvents equally defined them as usable but not recommended for chemical manufacturing, with the only exception being EtOAc that was classified as recommended.^34^ Conversions of 86–96% were obtained with the least polar solvents at 0.4≤a_w_≤0.8, whereas water‐saturated solvents generally showed very poor performance. Toluene at controlled a_w_ of 0.7 was a better solvent than *n*‐heptane for our system because of the higher volatility of the latter that complicates its use particularly in a flow apparatus. Conversely, *n‐*decane proved to be unsuitable due to its difficult removal from the final product by evaporation. Thus, full conversion was observed within 48 h reaction time using toluene as solvent (Figure 2 and Table S10). Future studies aimed at large scale implementation of this process must consider the replacement of toluene with recommended “greener” alternatives possessing similar physicochemical properties such as anisole, or 2‐methyltetrahydrofuran or 2,2,5,5‐tetramethyloxolane.^34^, ^35^ Notably, EziG^3^−Cv (ωTA from *Chromobacterium violaceum*) employed in toluene was found to be most active at a_w_≤0.2 (Figure 1C and Table S7). This finding indicates that different ωTAs operate in a neat organic environment at different optimal a_w_ values.

Next, the dependency of the reaction temperature was tested by applying EziG^3^−AsR−ωTA at 25 °C, 40 °C and 50 °C in toluene (controlled a_w_). Similar conversion trends were observed in the 0.2≤a_w_≤0.7 range, albeit conversions were lower at higher temperatures (Table S8 and S6), as was previously observed for the non‐immobilized ωTA.^26^ We then tested the possibility of reducing the equivalents of the amine donor for the reaction. Notably, an equimolar concentration of **1 a** and **2 b** (50 mM) was sufficient to reach 84±2% conversion in the organic solvent (Table S9). The maximum productivity using an equimolar concentration of **1 a** and **2 b** was obtained at 450 mM substrates concentration, yielding 76±1% conversion (equal to 344 mM, 52 mg of **1 b** (Table S11)). Finally, recycling of EziG^3^−AsR−ωTA for reaction cycles at substrate concentrations up to 400 mM was investigated. After each reaction cycle (72 h, using 1 eq. of amine donor **2 b**), the reaction mixture was separated from the biocatalyst and the conversion was measured by GC. The same batch of EziG^3^−AsR−ωTA biocatalyst was then re‐suspended in a fresh reaction mixture containing the reagents and another reaction cycle was initiated (Figure S2 and Table S12; for experimental details, see SI, section 4.9). At substrate concentrations of 300 mM and 400 mM, a significant drop in conversion was observed after the first reaction cycle; however, it resulted in an overall analytical product yield of 110 mg of **1 b** over four reaction cycles (TTN=13600). No evident enzyme leaching was detected under the applied reaction conditions and within the number of cycles that were run.

In general, we expected to obtain better system durability by implementing the immobilized biocatalysts in a packed‐bed flow reactor. Performing reactions in continuous flow has become a practical tool for the recycling of enzymes.^36^ In particular, packed bed flow reactors have received considerable attention in biocatalysis because they avoid additional separation steps and prevent enzyme deactivation caused by mechanical stirring in classical bioreactors.^37^ We previously reported the application of EziG^3^−AsR−ωTA in a packed‐bed reactor for the kinetic resolution of racemic amines in aqueous buffer, which proceeded without any detectable loss of enzymatic activity for five days.^26^

**Figure 3:**
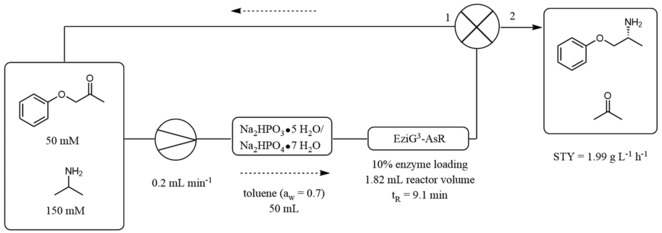
General loop set‐up for flow reactions in organic solvent with EziG^3^−AsR−ωTA at controlled a_w_. **(1)** The reaction mixture is directed back to the inlet of the flow reactor. **(2)** Product isolation. The apparatus comprises: a) a stainless steel column (20 cm×4 mm) filled with EziG^3^−AsR−ωTA (400 mg, enzyme loading: 10% w w^−1^) and Na_2_HPO_3_ ⋅ 5H_2_O/Na_2_HPO_4_ ⋅ 7H_2_O (600 mg, ratio 1:1 w w^−1^); b) a stainless steel pre‐column (5 cm×10 mm) packed with Na_2_HPO_3_ ⋅ 5H_2_O/Na_2_HPO_4_ ⋅ 7H_2_O (4 g, ratio 1:1 w w^−1^).

**Figure 4:**
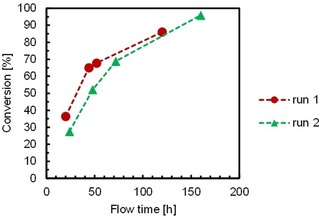
Continuous flow reaction with EziG^3^−AsR−ωTA in the transamination of **1 a** in toluene at controlled a_w_ (a_w_=0.7). Immobilization conditions: EziG^3^ (Fe Amber, 400 mg), AsR−ωTA (40 mg, 1.07 μmol, enzyme loading: 10% w w^−1^) in KPi buffer (10 mL, 100 mM, pH 8.0), 4 °C, 120 rpm; incubation time: 3 h. Reaction conditions: EziG^3^−AsR−ωTA (440 mg, enzyme loading: 10% w w^−1^), salt hydrate pairs (ca. 600 mg), toluene (a_w_=0.7), **1 a** (50 mM), **2 b** (150 mM), 25 °C; flow rate: 0.2 mL min^−1^. All reactions afforded >99% enantiomeric excess of the amine product.

We performed the transamination of **1 a** in an organic solvent in a continuous flow stainless steel packed‐bed reactor containing the immobilized ωTA (EziG^3^−AsR) mixed with hydrate salt pairs (ratio 2:3 w w^−1^) in order to ensure controlled a_w_ in the system (reactor volume: 1.82 mL, residence time, t_R_=9.1 min, flow rate: 0.2 mL min^−1^). The flow system was also equipped with a pre‐column containing hydrate salt pairs in order to pre‐equilibrate the organic solvent before entering the reactor. Thus, a solution of **1 a** (50 mM) and **2 b** (150 mM) in toluene (50 mL) was pumped through the system. In order to reduce flow times, the outlet of the flow reactor was connected to the inlet of the pump, thereby creating a loop (Figure 3). The flow reactor showed excellent performance in two independent experiments and the ratio between **1 a** and **1 b** gradually increased to ca. 70% conversion in 72 hours (STY=1.99 g L^−1^ h^−1^) and up to above 90% in 120 hours (Figure 4 and Table S13). In a third independent experiment, we observed a slight decrease in conversion over time (Table S13, entries 9–18). The addition of a fresh aliquot of IPAm (150 mM) did not significantly improve the performance of the flow reactor.

In order to prove the reproducibility of the transamination in the flow reactor, the set‐up was tested with 20 mM **1 a** and 100 mM **2 b** in repetitive cycles of 24 hours flow‐time each (Table S14, entries 1–5). In practice, the flow system was operated for 24 h at a constant flow rate (0.2 mL min^−1^, 20 mL reaction volume, 20 mM **1 a**) and the conversion was determined by GC (production of **1 b**: ca. 32–33 mg day^−1^). Then, a fresh reaction mixture was loaded and the flow reaction was continued for another 24 h. The process was continuously repeated for five days with no observable loss in performance and the pure amine product **1 b** was isolated in 84% isolated yield, 126 mg, >99% chemical purity and >99% *ee* (measured by GC). Notably, the flow reactor was still performing at 50% of its initial activity after four weeks of storage at 4 °C (Table S14, entries 6–7).

## Conclusion

We demonstrated high catalytic performance of ωTAs immobilized on controlled porosity metal‐ion affinity carriers (EziG) in neat organic solvents at controlled a_w_. A robust reaction system was developed using hydrate salt pairs for the optimization and control of c_w_ (and hence a_w_) in non‐polar solvents. High catalytic activity was obtained by optimizing the system in terms of immobilization buffer, carrier material and reaction solvent. Significant improvements in productivity were achieved when applying higher substrate concentrations and only one equivalent of amine donor was required in the reaction to reach conversions above 80% (with >99% *ee*). Finally, practical applicability was demonstrated in a continuous flow packed‐bed reactor producing a chiral amine with excellent productivity and without observable loss in catalytic performance over several days of operation. This work demonstrates the potential of continuous flow biocatalysis in neat organic solvents using selective immobilization of ωTAs on controlled porosity metal‐ion affinity carriers.

## Experimental Section

### Chemicals and Carrier Materials

2‐propylamine, phenoxypropan‐2‐one and pyridoxal‐5′‐phosphate (PLP) were purchased from Sigma‐Aldrich (Steinheim, Germany). The following EziG enzyme carrier material was provided by EnginZyme AB (Solna, Sweden): EziG^1^ (Fe Opal), EziG^2^ (Fe Coral) and EziG^3^ (Fe Amber). Further details for equipment and analytical determination are available in SI, section 2. All reaction solvents were degassed before use. All of the water‐equilibrated solvents were prepared by shaking hydrate salt pairs in the organic solvent for 1 h at RT.

### Expression and Purification of ωTAs

C‐terminal His‐tagged (*R*)‐selective ωTA from *Arthrobacter sp*. (AsR−ωTA)^28^ and N‐terminal His‐tagged (*S*)‐selective ωTA from *Chromobacterium violaceum* (Cv−ωTA)29a, 29b, 34b were expressed and purified as previously reported.^26^

### Immobilization of ωTAs on EziG Carrier Materials

On analytical scale, EziG carrier material (20±0.2 mg) was cooled down in an ice bath and suspended in an immobilization buffer (KPi, 1 mL, 100 mM, pH 8.0) supplemented with PLP (0.1 mM). Purified ω‐TA (2 mg, equal to 10% w w^−1^, enzyme loading to support material) was added to the suspension. The mixture was shaken on an orbital shaker (120 rpm) for 3 h at 4 °C. Small aliquots from the aqueous phase (20 μL) were taken before and after the immobilization procedure; their concentrations were determined using the Bradford assay (see SI section 2.2 for details). Once full immobilization was obtained, the immobilized enzyme was let to sediment and the buffer was removed by pipetting.

The same procedure was also followed for immobilization at a larger scale, typically using 40 mg of purified ωTA and 400 mg of EziG carrier material.

### Analytical Scale Reactions in Organic Solvents with Immobilized ωTAs

EziG‐immobilized ωTA (total mass 20 mg, 10% w w^−1^ enzyme loading to support material) and hydrate salts (Na_2_HPO_x_ ⋅ yH_2_O/Na_2_HPO_z_ ⋅ wH_2_O, total mass 20 mg, 1:1, w w^−1^) were suspended in EtOAc (1 mL, at controlled a_w_) and shaken for 15 min (900 rpm, thermomixer). The immobilized enzyme was allowed to sediment and the solvent was removed by pipetting. The immobilized enzyme with hydrate salt pairs was suspended in EtOAc and the process was repeated twice. The immobilized enzyme with hydrate salts was washed with reaction solvent (1 mL, at fixed a_w_). The immobilized enzyme was allowed to sediment and the solvent was removed by pipetting. Reaction solvent (900 μL, at fixed a_w_) was added. A 10‐fold stock of **2 b** was prepared in the reaction solvent and added (final concentration: 150 mM, unless otherwise indicated). Finally, **1 a** (6.89 μL, 0.05 mmol, final concentration: 50 mM) was added and the reaction vials were shaken in an upright position (900 rpm, thermomixer) for 72 h at 25 °C. Work‐up was performed by drying over MgSO_4_ and analysis was performed by injection on GC with an achiral column (see SI, Section 2.3 for details on analytical equipment and determination). For determination of the enantiomeric excess, the samples were derivatized using 4‐dimethylaminopyridine in acetic anhydride (final concentration: 5 mg mL^−1^) for 30 min (170 rpm, RT). Samples were quenched by the addition of water (500 μL) and shaken for 30 min (170 rpm, RT). After centrifugation, the organic layer was dried over MgSO_4_ and analyzed by GC with a chiral column (see SI, Section 2.3 for details on analytical equipment and determination).

### Flow Reactions in Organic Solvents with Immobilized ωTA

EziG^3^−AsR (total mass 440 mg, 10% w w^−1^ enzyme loading to support material) and a hydrate salt pair (Na_2_HPO_3_ ⋅ 5H_2_O/Na_2_HPO_4_ ⋅ 7H_2_O, total mass 600 mg, 1:1 w w^−1^) were suspended in EtOAc (10 mL, a_w_=0.7) and shaken for 15 min (120 rpm, orbital shaker). The immobilized enzyme was allowed to sediment and the solvent was removed by pipetting. The immobilized enzyme with hydrate salts was suspended in EtOAc and the process was repeated four times. The immobilized enzyme with hydrate salts was washed with reaction solvent (10 mL, a_w_=0.7). The immobilized enzyme was allowed to sediment and the solvent was removed by pipetting. The immobilized enzyme with hydrate salts was packed into a stainless steel column (20 cm×0.4 cm, 1.82 mL), which was attached to a Dionex HPLC pump. A stainless steel pre‐column (5 cm×1 cm) was filled with hydrate salts (4 g, Na_2_HPO_3_ ⋅ 5H_2_O/Na_2_HPO_4_ ⋅ 7H_2_O, 1:1 w w^−1^) and attached between the pump and the flow reactor. A solution of **1 a** (375 μL, 50 mM final concentration) and **2 b** (640 μL, 150 mM final concentration) in toluene (50 mL, a_w_=0.7) was pumped through the flow reactor (equipped with pre‐column) at a rate of 0.2 mL min^−1^. The flow reactor outlet was directed back into the reaction mixture as shown in Figure 3. Conversions were determined by GC equipped with an achiral column (see SI, Section 2.3 for details on analytical equipment and determination). A work‐up was performed by evaporation of the reaction solvent and the residue was dissolved in 2 M HCl (12 mL). The aqueous layer was extracted with MTBE (3×10 mL) and then basified to pH 12 with 10 M KOH and extracted again with MTBE (3×10 mL). The second organic layer was dried over MgSO_4_ and evaporated to dryness, yielding the amine product **1 b** in high purity (>99% purity by GC, >99% *ee*).

## Supporting information

As a service to our authors and readers, this journal provides supporting information supplied by the authors. Such materials are peer reviewed and may be re‐organized for online delivery, but are not copy‐edited or typeset. Technical support issues arising from supporting information (other than missing files) should be addressed to the authors.

Supplementary
