# Supplementary material for: Continuous Flow Bioamination of Ketones in Organic Solvents at Controlled Water Activity using Immobilized ω‐Transaminases
Source: Adv Synth Catal. 2020 Feb 17;362(9):1858–67. doi: 10.1002/adsc.201901274 (PMC7217232; doi:10.1002/adsc.201901274)
Supplement: Supplementary file 1 — Supplementary [file ADSC-362-1858-s001.pdf]

Advanced Synthesis & Catalysis

Supporting Information

Continuous Flow Bioamination of Ketones in Organic Solvents at Controlled Water Activity using Immobilized ω-Transaminases

Wesley Böhmer, Alexey Volkov, Karim Engelmark Cassimjee, and Francesco G. Mutti*© 2020 The Authors. Published by Wiley-VCH Verlag GmbH & Co. KGaA. This is an open access article under the terms of the Creative Commons Attribution License, which permits use, distribution and reproduction in any medium, provided the original work is properly cited.

Supporting Information for:

Enhancing the Applicability of the Bioamination of Ketones in Neat Organic Solvents using ω-Transaminases as Immobilized Biocatalysts in Continuous Flow

Wesley Böhmer,[a] Alexey Volkov,[b] Karim Engelmark Cassimjee,[b] Francesco G. Mutti [a,*]

[a] Van 't Hoff Institute for Molecular Sciences, HIMS-Biocat, University of Amsterdam, Science Park 904, 1098 XH Amsterdam, The Netherlands

[b] EnginZyme AB, Tomtebodavägen 6, 171 65 Solna, Sweden

* Corresponding author: f.mutti@uva.nl

Table of Contents

1. Abbreviations 2
2. General information 3
2.1 Equipment 3
2.2 Bradford assay 3
2.3 Analytics 4
2.4 EziG product specifications 4
3. Calculations and terminology 4
3.1 Immobilization yield 4
3.2 Water activity 5
3.3 Calculation of amine conversion 6
3.4 Reaction rate in flow reactors 7
4. Results 8
4.1 Effect of the pH of the immobilization buffer on the activity of immobilized ωTA in organic solvent as reaction medium 8
4.2 Amination in organic solvents catalyzed by ω-transaminases immobilized on three types of EziG carrier materials 8
4.3 Testing different reaction solvents with EziG-immobilized transaminase 10
4.4 Activity of EziG-immobilized Cv-ωTA at controlled α_w 11
4.5 Activity of EziG-immobilized AsR-ωTA at different temperatures 12
4.6 Decrease of the amine donor concentration for the amination catalyzed by EziG-immobilized AsR-ωTA and in organic solvent as reaction medium 13
4.7 Time study for the amination catalyzed by EziG-immobilized AsR-ωTA in toluene as reaction medium 14
4.8 Amination catalyzed by EziG-immobilized AsR-ωTA in toluene as reaction medium and applying higher substrate concentration 15
4.9 Recyclability of EziG-immobilized AsR-ωTA in toluene 16
4.10 Continuous flow experiments 17
5. References 20

1. Abbreviations

AsR-ωTA	(*R*)-selective ω-transaminase from *Arthrobacter* species
α_w	water activity
Cv-ωTA	(*S*)-selective ω-transaminase from *Chromobacterium violaceum*
CPG	controlled porosity glass
c_w	water content
DMAP	dimethylaminopyridine
DMSO	dimethylsulfoxide
EtOAc	ethyl acetate
$EziG^3$-AsR	AsR-ωTA immobilized on $EziG^3$ Fe Amber
$EziG^3$-Cv	Cv-ωTA immobilized on $EziG^3$ Fe Amber
GC	gas chromatography
HEPES	4-(2-hydroxyethyl)-1-piperazineethanesulfonic acid
IPAm	isopropylamine
KOH	potassium hydroxide
$MgSO_4$	magnesium sulfate
MOPS	3-(*N*-morpholino)propanesulfonic acid
n.a.	not applicable
n.d.	not determined
PLP	pyridoxal 5'-phosphate
RT	room temperature

2. General information

2.1 Equipment

For the immobilization of enzymes on carrier material, a C-star orbital shaker no. 12846016 (Thermo Fisher Scientific, UK) was used. Biorad protein assay dye reagent concentrate was purchased from Carl Roth (Karlsruhe, Germany). Biotransformations were performed in an Eppendorf Thermomixer compact 5350 (Germany). Continuous flow experiments were performed with a Dionex P680 HPLC pump unit (Thermo Fischer Scientific, UK).

Table S1. List of compounds.

entry	no.	name	chemical structure
1	**1a**	phenoxypropan-2-one	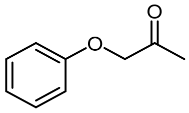
2	**1b**	phenoxypropan-2-amine	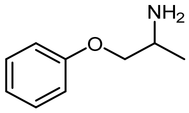
3	imine-**1a**/**2b**	*N*-isopropyl-1-phenoxypropan-2-imine	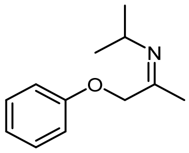
4	imine-**1b**/**2a**	*N*-(1-phenoxypropan-2-yl)propan-2-imine	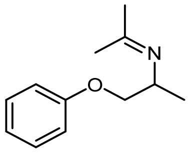
5	**2b**	2-propylamine	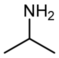
6	**2a**	acetone	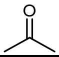

2.2 Bradford assay

Concentrated Biorad protein assay dye reagent was diluted 5-fold with MilliQ water and filtered over a paper filter. The stock solution was freshly prepared before use and kept in the dark at 4 °C. Albumin calibration was performed in the standard range of 200-1000 µg mL^{-1} protein. For lower protein concentration (<25 µg mL^{-1}), the low-concentration assay of 1-20 µg mL^{-1} was used. Samples were prepared by mixing 980 µL stock solution and 20 µL protein sample (for low-concentration assay: 800 µL stock and 200 µL protein sample) followed by incubation for 5-10 minutes at RT. Absorption at the wavelength of 595 nm was measured and plotted against the protein concentration. Diluted enzyme samples were then measured in the same fashion in order to determine their concentration.

2.3 Analytics

Conversions were determined by GC using a 7890A GC system (Agilent Technologies), equipped with a FID detector, using H_2 as carrier gas, and a DB1701 column from Agilent (30 m, 250 µm, 0.25 µm). The enantiomeric excess of the derivatized amines was measured using a ChiraSil DEX-CB column from Agilent (25 m, 320 µm, 0.25 µm).

HP-5 method: constant pressure 4 psi, T injector 250 °C, split ratio 30:1, T initial 60 °C, hold 0 min; gradient 5 °C/min up to 150 °C, hold 1 min, gradient 10 °C/min up to 250 °C, hold 1 min.

ChiraSil DEX-CB method: constant flow 1.4 mL/min, T injector 250 °C, split ratio 20:1, T initial 100 °C, hold 2 min; gradient 1 °C/min up to 130 °C, hold 5 min; gradient 10 °C/min up to 170 °C, hold 10 min.; gradient 10 °C/min up to 180 °C, hold 1 min.

Table S2. GC retention time of reference compounds.

entry	compound	retention time [min]	GC column
1	**1a**	12.7	HP-5
2	**1b**	13.9	HP-5
3	imine-**1a/2b**	17.6	HP-5
4	imine-**1b/2a**	18.5	HP-5
5	(*R*)-**1b**[1]	44.7	ChiraSil DEX-CB
6	(*S*)-**1b**[1]	43.9	ChiraSil DEX-CB

[1] Upon derivatization to acetoamido.

2.4 EziG product specifications

Table S3. EziG product specifications: particle size 75-125 µm (100-300 mesh), chelated Fe^{3+} >10 µmol/g.

entry	product	surface	pore diameter [nm]	pore volume [mL/g]	bulk density [g/mL]	pH range	Lot#
1	EziG[1] Fe Opal	Directly derivatized hydrophilic glass	50±5	ca. 1.8	0.25-0.32	5-10	MR010716
2	EziG[2] Fe Coral	Hydrophobic polymer	30±5	ca. 1.8	0.21-0.25	5-10	MR011916
3	EziG[3] Fe Amber	Semi-hydrophobic copolymer	30±5	ca. 1.8	0.21-0.25	5-10	EziG-130

3. Calculations and terminology

3.1 Immobilization yield

In order to determine how much of the enzyme is immobilized during the process, a Bradford assay (UV absorption at 595 nm, section 2.2) was performed before ($A_{595\ initial}$) and after the immobilization process ($A_{595\ final}$) for calculating the amount of enzyme bound to the beads, i.e., the immobilization yield (Equation 1).

Equation 1

$$immobilization\ yield\ [\%] = \frac{(A_{595\,final} - A_{595\,initial})}{A_{595\,initial}} \times 100\%$$

3.2 Water activity

For obtaining reaction solvents with a controlled α_w, organic solvents were stirred for 1 hour in presence of sodium dibasic phosphate hydrate salt pairs (1:1 w w^{-1}, ratio of hydrates). Previous studies indicate this time to be sufficient for α_w to reach an equilibrium between the organic and the solid phase.[1] Equilibrium α_w for organic solvents in presence of Na_2HPO_4/Na_2HPO_4•$2H_2O$, Na_2HPO_4•$2H_2O$/ Na_2HPO_4•$7H_2O$, and Na_2HPO_4•$7H_2O$/ Na_2HPO_4•$12H_2O$ was previously determined to be α_w = 0.16, α_w = 0.59, and α_w = 0.80 respectively.[1-2] In our studies we prepared also Na_2HPO_4•$2H_2O$/ Na_2HPO_3•$5H_2O$ and Na_2HPO_3•$5H_2O$/ Na_2HPO_4•$7H_2O$ with 0.16 < α_w < 0.59 and 0.59 < α_w < 0.80 respectively. For the sake of simplicity, we indicated α_w of Na_2HPO_4•$2H_2O$/ Na_2HPO_3•$5H_2O$ and Na_2HPO_3•$5H_2O$/ Na_2HPO_4•$7H_2O$ as α_w = 0.4, and α_w = 0.7 respectively (Table S4).

Table S4. Water activity of organic solvents fixed by using hydrate salt pairs. Combinations of different hydrate salt pairs enable to obtain different water activity. Ratio of hydrate salt pairs: 1:1 (w w^{-1}).

entry	hydrate salt pairs	α_w	references
1	Na_2HPO_4/Na_2HPO_4•$2H_2O$	0.16	[1]
2	Na_2HPO_4•$2H_2O$/ Na_2HPO_3•$5H_2O$	~0.4	extrapolated
3	Na_2HPO_4•$2H_2O$/ Na_2HPO_4•$7H_2O$	0.61	[2]
4	Na_2HPO_3•$5H_2O$/ Na_2HPO_4•$7H_2O$	~0.7	extrapolated
5	Na_2HPO_4•$7H_2O$/ Na_2HPO_4•$12H_2O$	0.80	[2]
6	pure water or water-saturated	1.00	As defined in thermodynamics.

3.3 Calculation of amine conversion

The reactions with immobilized ωTAs in organic solvents were analyzed by GC (see section 2.3 for details on analytical equipment and methods). Retention times of observed compounds are listed in Table S2. Apart from the substrate **1a** and product **1b**, corresponding imines (imine-**1a/2b** and imine-**1b/2a**, see Table S1) were observed. It is important to remark that imine formation is due to the spontaneous and reversible addition of amines to ketones that are present in the same reaction mixture. However, the amine product could always be quantitatively isolated upon proper work-up of the organic phase.

The GC response of the observed imines was determined and compared to those of **1a** and **1b**. We performed GC calibration using 20 mM, 50 mM, or 100 mM **1a** (or **1b**) by measuring the GC peak area of the standard samples. In a second set of standard samples, we dissolved **1a** (20 mM, 50 mM, or 100 mM) in neat **2b** as well as **1b** (20 mM, 50 mM, or 100 mM) in neat **2a** in order to form the imines in solution in nearly quantitative amounts (>95%). The GC peak area of each standard sample was plotted against the concentration of analyte (Figure S1). Notably, no significant difference in response was observed among **1a**, **1b** and imines **1a/2b** and **1b/2a**. Following this observation we calculated the conversion to **1b** in the reaction mixture containing imine-**1a/2b** and/or imine-**1b/2a** by adding up GC areas of **1a** with those of imine-**1a/2b** and GC areas of **1b** with those of imine-**1b/2a** in order to obtain the actual reaction conversion:

Equation 3:

$$final\ conversion\ to\ \mathbf{1b}\ [\%] = \frac{GC\ area\ \mathbf{1b} + GC\ area\ imine\ \mathbf{1b}/\mathbf{2a}}{(GC\ area\ \mathbf{1a} + GC\ area\ \mathbf{1b} + GC\ area\ imine\ \mathbf{1a}/\mathbf{2b} + GC\ area\ \mathbf{1b}/\mathbf{2a})} * 100\%$$

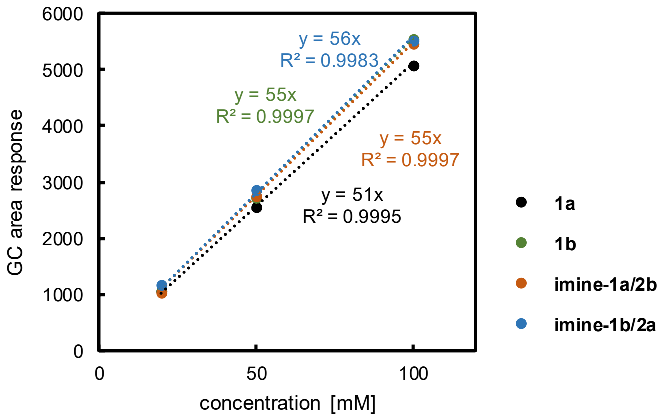

Figure S1. GC area response of substrates and products. The GC area response of 20 mM, 50 mM, or 100 mM of **1a** (or **1b**) was measured. Imine formation was generated by dissolving **1a** in neat **2b** (imine-**1a/2b**) or **1b** in neat **2a** (imine-**1b/2a**).

3.4 Reaction rate in flow reactors

In flow reactors, several parameters relate to the reaction rate. An important parameter is space velocity (SV, in units of reciprocal time), which is defined by the volumetric flow rate of the reactant stream (V_o, specified at the inlet conditions of temperature and pressure with zero conversion), and the catalyst volume (V_c). Often catalyst volume (V_c) is equally related to the reactor volume (V_r), which depends on the packing density of the catalyst particles.

Equation 4:

$$SV\ (space\ velocity) = \frac{V_o}{V_r}$$

Space time (τ, in units of time) is the inverse of space velocity and it gives the time required to process one reactor volume:

Equation 5:

$$\tau\ (space\ time) = \frac{1}{SV} = \frac{V_r}{V_o}$$

The space time yield (STY) refers to the quantity of product produced per quantity of catalyst per unit time. If the catalyst is well-packed in the full reactor, then the catalyst volume (V_c) can be equated to the reactor volume (V_r).

Equation 6:

$$STY\ (space\ time\ yield) = \frac{product\ produced\ [g]}{(V_r\ \times time)}$$

Calculation of space-time yield for the flow process described in the manuscript:

$$production\ of\ \mathbf{1b}\ [mg] = 0.69\ (conv.) * 0.05\ \frac{mol}{L} * 0.05\ L * 151.2 \frac{g}{mol} = 0.261\ g$$

$$STY = \frac{\mathbf{1b}\ [g]}{(reactor\ volume\ [L] * reaction\ time\ [h])} = \frac{0.261\ [g]}{(0.00182\ [L] \times 72\ [h])} = 1.99\ g\ L^{-1}h^{-1}$$

4. Results

Preparation of EziG-immobilized ω-transaminases was optimized for application in neat organic solvents in terms of type of immobilization buffer, buffer pH, water-equilibrated solvent, reaction solvent and α_w. AsR-ωTA was chosen as a model enzyme. As indicated in the experiments in this section, either the immobilization conditions or the reaction solvents were changed. Unless stated otherwise, the reaction conditions for the reductive amination assay were as depicted in Scheme 1 (main manuscript).

4.1 Effect of the pH of the immobilization buffer on the activity of immobilized ωTA in organic solvent as reaction medium

AsR-ωTA was immobilized (analytical scale) in KPi buffer (100 mM) at 4 °C. AsR-ωTA (2 mg, 54 nmol) was diluted in 1 mL buffer containing PLP (0.1 mM) and EziG carrier material (20 mg) was added. The suspension was shaken (120 rpm) for 3 hours after which aliquots (20 µL) of the buffer solution were taken and the protein concentration was determined by Bradford assay (section 2.2). The immobilized ωTA was water-equilibrated with EtOAc in the presence of $Na_2HPO_3 \cdot 5H_2O/Na_2HPO_4 \cdot 7H_2O$ (ca. 25 mg, ratio 1:1 w w^{-1}, see experimental section — main manuscript). The reaction was performed in toluene (α_w = 0.7) with 50 mM **1a** and 150 mM **2b** at 25 °C (see experimental section — main manuscript). Conversions were determined by GC (see section 2.3).

4.2 Amination in organic solvents catalyzed by ω-transaminases immobilized on three types of EziG carrier materials

AsR-ωTA was immobilized (analytical scale) under standard conditions (see experimental section — main manuscript). The immobilization was performed in KPi buffer (100 mM, pH 8) at 4 °C. AsR-ωTA (2 mg, 54 nmol) was diluted in 1 mL buffer containing PLP (0.1 mM) and EziG carrier material (20 mg) was added. The suspension was shaken (120 rpm) for 1 hour in the case of samples supplied with EziG[1] Fe Opal, and 3 hours in the case of samples supplied EziG[2] Fe Coral or EziG[3] Fe Amber. Aliquots (20 µL) of the buffer solution were taken and the remaining protein concentration was determined by Bradford assay (section 2.2). The immobilized ωTA was water-equilibrated with EtOAc in the presence of $Na_2HPO_3 \cdot 5H_2O/Na_2HPO_4 \cdot 7H_2O$ (ca. 25 mg, ratio 1:1 w w^{-1}, see experimental section — main manuscript). The reaction was performed in toluene (α_w = 0.7) with 50 mM **1a** and 150 mM **2b** at 25 °C (see experimental section — main manuscript). Conversions were determined by GC (see section 2.3).

Table S5. AsR-ωTA immobilized on EziG carrier materials and tested in organic solvent at controlled α_w. Immobilization conditions: EziG[1] (Fe Opal, 20 mg, lot#MR010716) or EziG[2] (Fe Coral, 20 mg, lot#MR011916) or EziG[3] (Fe Amber, 20 mg, lot#EziG-130), AsR-ωTA (2 mg, 54 nmol, enzyme loading: 10% w w^{-1}), KPi buffer (1 mL, 100 mM, pH 8.0), PLP (0.1 mM), 4 °C, 120 rpm, incubation time: 3 h. Reaction conditions: EziG[1]-AsR or EziG[2]-AsR or EziG[3]-AsR (22 mg, enzyme loading: 10% w w^{-1}), hydrate salt pair (ca. 25 mg), toluene (reaction volume: 1 mL, α_w as specified), **2b** (150 mM), **1a** (50 mM), 25 °C, 900 rpm, reaction time: 72 h.

entry	carrier type	α_w	hydrate salts	conversion [%][1]
1	EziG[1] Fe Opal	0.2	Na_2HPO_4/Na_2HPO_4•$2H_2O$	80±10
2		0.4	Na_2HPO_4•$2H_2O$/Na_2HPO_3•$5H_2O$	81±9
3		0.6	Na_2HPO_4•$2H_2O$/Na_2HPO_4•$7H_2O$	84±7
4		0.7	Na_2HPO_3•$5H_2O$/Na_2HPO_4•$7H_2O$	98±0
5		0.8	Na_2HPO_4•$7H_2O$/Na_2HPO_4•$12H_2O$	70±2
6		water-saturated	n.a.[2]	15±1
7	EziG[2] Fe Coral	0.2	Na_2HPO_4/Na_2HPO_4•$2H_2O$	22±4
8		0.4	Na_2HPO_4•$2H_2O$/Na_2HPO_3•$5H_2O$	25±2
9		0.6	Na_2HPO_4•$2H_2O$/Na_2HPO_4•$7H_2O$	65±10
10		0.7	Na_2HPO_3•$5H_2O$/Na_2HPO_4•$7H_2O$	95±0
11		0.8	Na_2HPO_4•$7H_2O$/Na_2HPO_4•$12H_2O$	49±5
12		water-saturated	n.a.[2]	7±0
13	EziG[3] Fe Amber	0.2	Na_2HPO_4/Na_2HPO_4•$2H_2O$	75±16
14		0.4	Na_2HPO_4•$2H_2O$/Na_2HPO_3•$5H_2O$	96±1
15		0.6	Na_2HPO_4•$2H_2O$/Na_2HPO_4•$7H_2O$	87±1
16		0.7	Na_2HPO_3•$5H_2O$/Na_2HPO4•$7H_2O$	98±0
17		0.8	Na_2HPO_4•$7H_2O$/Na_2HPO_4•$12H_2O$	87±0
18		water-saturated	n.a.[2]	3±0

[1] Values are depicted as actual conversion to amine product with standard deviation over three independent experiments. [2] Not applicable.

4.3 Testing different reaction solvents with EziG-immobilized transaminase

AsR-ωTA was immobilized (analytical scale) under standard conditions (see experimental section ─ main manuscript). The immobilized ωTA was water-equilibrated with EtOAc in the presence of hydrate salts (ca. 25 mg, ratio 1:1 w w^{-1}, see experimental section ─ main manuscript and Table S6). The reaction was performed in different reaction solvents (Table S6) with 50 mM **1a** and 150 mM **2b** at 25 °C. Conversions were determined by GC (see section 2.3).

Table S6. Study of $EziG^3$-AsR applied in different reaction solvents at controlled $α_w$. Immobilization conditions: $EziG^3$ (Fe Amber, 20 mg, lot#EziG-130), AsR-ωTA (2 mg, 54 nmol, enzyme loading: 10% w w^{-1}), KPi buffer (1 mL, 100 mM, pH 8.0), PLP (0.1 mM), 4 °C, 120 rpm, incubation time: 3 h. Reaction conditions: $EziG^3$-AsR (22 mg, enzyme loading: 10% w w^{-1}), hydrate salt pair (ca. 25 mg), reaction solvent (reaction volume: 1 mL, $α_w$ as specified), **2b** (150 mM), **1a** (50 mM), 25 °C, 900 rpm, reaction time: 72 h.

entry	reaction solvent	log P	$α_w$	hydrate salts	conversion [%][1]
1	EtOAc	0.7	0.2	$Na_2HPO_4/Na_2HPO_4{\bullet}2H_2O$	8±5
2			0.4	$Na_2HPO_4{\bullet}2H_2O/Na_2HPO_3{\bullet}5H_2O$	45±6
4			0.7	$Na_2HPO_3{\bullet}5H_2O/Na_2HPO_4{\bullet}7H_2O$	66±5
5			0.8	$Na_2HPO_4{\bullet}7H_2O/Na_2HPO_4{\bullet}12H_2O$	69±2
6			water-saturated	n.a.[2]	14±1
7	MTBE	0.9	0.2	$Na_2HPO_4/Na_2HPO_4{\bullet}2H_2O$	6±1
8			0.4	$Na_2HPO_4{\bullet}2H_2O/Na_2HPO_3{\bullet}5H_2O$	63±2
10			0.7	$Na_2HPO_3{\bullet}5H_2O/Na_2HPO_4{\bullet}7H_2O$	73±3
11			0.8	$Na_2HPO_4{\bullet}7H_2O/Na_2HPO_4{\bullet}12H_2O$	93±1
12			water-saturated	n.a.[2]	30±8
13	toluene	2.5	0.2	$Na_2HPO_4/Na_2HPO_4{\bullet}2H_2O$	53±9
14			0.4	$Na_2HPO_4{\bullet}2H_2O/Na_2HPO_3{\bullet}5H_2O$	93±1
16			0.7	$Na_2HPO_3{\bullet}5H_2O/Na_2HPO_4{\bullet}7H_2O$	95±0
17			0.8	$Na_2HPO_4{\bullet}7H_2O/Na_2HPO_4{\bullet}12H_2O$	79±4
18			water-saturated	n.a.[2]	72±2
19	n-heptane	4.0	0.2	$Na_2HPO_4/Na_2HPO_4{\bullet}2H_2O$	47±12
20			0.4	$Na_2HPO_4{\bullet}2H_2O/Na_2HPO_3{\bullet}5H_2O$	86±6
22			0.7	$Na_2HPO_3{\bullet}5H_2O/Na_2HPO_4{\bullet}7H_2O$	96±1
23			0.8	$Na_2HPO_4{\bullet}7H_2O/Na_2HPO_4{\bullet}12H_2O$	94±1
24			water-saturated	n.a.[2]	34±1
25	decane	5.6	0.2	$Na_2HPO_4/Na_2HPO_4{\bullet}2H_2O$	73±4
26			0.4	$Na_2HPO_4{\bullet}2H_2O/Na_2HPO_3{\bullet}5H_2O$	93±0
28			0.7	$Na_2HPO_3{\bullet}5H_2O/Na_2HPO_4{\bullet}7H_2O$	94±0
29			0.8	$Na_2HPO_4{\bullet}7H_2O/Na_2HPO_4{\bullet}12H_2O$	93±1
30			water-saturated	n.a.[2]	2±0

[1] Values are depicted as actual conversion to amine product with standard deviation over three independent experiments. [2] Not applicable.

4.4 Activity of EziG-immobilized Cv-ωTA at controlled α_w

Cv-ωTA was immobilized (analytical scale) under standard conditions (see experimental section — main manuscript). The immobilized ωTA was water-equilibrated with EtOAc in the presence of hydrate salts (ca. 25 mg, ratio 1:1 w w^{-1}, see experimental section — main manuscript and Table S7). The reaction was performed in toluene (α_w as specified, Table S7) with 50 mM **1a** and 150 mM **2b** at 25 °C. Conversions were determined by GC (see section 2.3).

Table S7. Study on $EziG^3$-Cv applied in toluene at controlled α_w. Immobilization conditions: $EziG^3$ (Fe Amber, 20 mg, lot#EziG-130), Cv-ωTA (2 mg, 38 nmol, enzyme loading: 10% w w^{-1}), KPi buffer (1 mL, 100 mM, pH 8.0), PLP (0.1 mM), 4 °C, 120 rpm, incubation time: 3 h. Reaction conditions: $EziG^3$-Cv (22 mg, enzyme loading: 10% w w^{-1}), hydrate salt pair (ca. 25 mg), reaction solvent (reaction volume: 1 mL, α_w as specified), **2b** (150 mM), **1a** (50 mM), 25 °C, 900 rpm, reaction time: 72 h.

entry	α_w	$EziG^3$-Cv conversion [%][1]
1	0.2	85±5
2	0.4	57±2
3	0.6	3±3
4	0.7	44
5	0.8	1
6	water-saturated	0

[1] Values are depicted as actual conversion to amine product with standard deviation over three independent experiments when applicable.

4.5 Activity of EziG-immobilized AsR-ωTA at different temperatures

AsR-ωTA was immobilized (analytical scale) under standard conditions (see experimental section — main manuscript). The immobilized ωTA was water-equilibrated with EtOAc in the presence of hydrate salts (ca. 25 mg, ratio 1:1 w w^{-1}, see experimental section — main manuscript and Table S8). The reaction was performed in toluene (α_w as specified, Table S8) with 50 mM **1a** and 150 mM **2b** at 40 °C or 50 °C. Conversions were determined by GC (see section 2.3).

Table S8. Temperature influence on performance of $EziG^3$-AsR at controlled α_w. Immobilization conditions: $EziG^3$ (Fe Amber, 20 mg, lot#EziG-130), AsR-ωTA (2 mg, 54 nmol, enzyme loading: 10% w w^{-1}), KPi buffer (1 mL, 100 mM, pH 8.0), PLP (0.1 mM), 4 °C, 120 rpm, incubation time: 3 h. Reaction conditions: $EziG^3$-AsR (22 mg, enzyme loading: 10% w w^{-1}), hydrate salt pair (ca. 25 mg), toluene (reaction volume: 1 mL, α_w as specified), **2b** (150 mM), **1a** (50 mM), temperature: as specified, 900 rpm, reaction time: 72 h.

entry	reaction temperature [°C]	α_w	hydrate salts	conversion [%][1]
1	40 °C	0.2	$Na_2HPO_4/Na_2HPO_4{\cdot}2H_2O$	13±8
2		0.4	$Na_2HPO_4{\cdot}2H_2O/Na_2HPO_3{\cdot}5H_2O$	76±4
3		0.6	$Na_2HPO_4{\cdot}2H_2O/Na_2HPO_4{\cdot}7H_2O$	37±9
4		0.7	$Na_2HPO_3{\cdot}5H_2O/Na_2HPO_4{\cdot}7H_2O$	80±2
5	50 °C	0.2	$Na_2HPO_4/Na_2HPO_4{\cdot}2H_2O$	7±2
6		0.4	$Na_2HPO_4{\cdot}2H_2O/Na_2HPO_3{\cdot}5H_2O$	58±32
7		0.6	$Na_2HPO_4{\cdot}2H_2O/Na_2HPO_4{\cdot}7H_2O$	17±2
8		0.7	$Na_2HPO_3{\cdot}5H_2O/Na_2HPO_4{\cdot}7H_2O$	64±6

[1] Values are depicted as actual conversion to amine product with absolute difference between two independent experiments.

4.6 Decrease of the amine donor concentration for the amination catalyzed by EziG-immobilized AsR-ωTA and in organic solvent as reaction medium

AsR-ωTA was immobilized (analytical scale) under standard conditions (see experimental section — main manuscript). The immobilized ωTA was water-equilibrated with EtOAc in the presence of hydrate salts (ca. 25 mg, ratio 1:1 w w^{-1}, see experimental section — main manuscript and Table S9). The reaction was performed in toluene (α_w as specified, Table S9) with 50 mM **1a** and 50 mM, 100 mM, or 150 mM **2b** at 25 °C. Conversions were determined by GC (see section 2.3).

Table S9. Transamination of **1a** catalyzed by $EziG^3$-AsR in organic solvent with 1, 2, or 3 equivalents **2b**. Immobilization conditions: $EziG^3$ (Fe Amber, 20 mg, lot#EziG-130), AsR-ωTA (2 mg, 54 nmol, enzyme loading: 10% w w^{-1}), KPi buffer (1 mL, 100 mM, pH 8.0), PLP (0.1 mM), 4 °C, 120 rpm, incubation time: 3 h. Reaction conditions: $EziG^3$-AsR (22 mg, enzyme loading: 10% w w^{-1}), $Na_2HPO_3 \cdot 5H_2O$/ $Na_2HPO_4 \cdot 7H_2O$ (ca. 25 mg), toluene (reaction volume: 1 mL, α_w= 0.7), **2b** (concentration as specified), **1a** (50 mM), 25 °C, 900 rpm, reaction time: 72 h.

entry	2b [mM]	equiv.	conversion [%][1]	*ee*% (*R*)
1	50	1	84±2	>99
2	100	2	98±0	>99
3	150	3	97±0	>99

[1] Values are depicted as actual conversion to amine product with standard deviation over three independent experiments.

4.7 Time study for the amination catalyzed by EziG-immobilized AsR-ωTA in toluene as reaction medium

AsR-ωTA was immobilized (analytical scale) under standard conditions (see experimental section ─ main manuscript). The immobilized ωTA was water-equilibrated with EtOAc in the presence of hydrate salts (ca. 25 mg, ratio 1:1 w w^{-1}, $Na_2HPO_3 \cdot 5H_2O/Na_2HPO_4 \cdot 7H_2O$). The reaction was performed in toluene (α_w = 0.7, Table S10) with 50 mM **1a** and 150 mM **2b** at 25 °C. Conversions were determined by GC (see section 2.3).

Table S10. Time study of $EziG^3$-AsR applied in toluene (α_w = 0.7) at 25 °C. Immobilization conditions: $EziG^3$ (Fe Amber, 20 mg, lot#EziG-130), AsR-ωTA (2 mg, 54 nmol, enzyme loading: 10% w w^{-1}), KPi buffer (1 mL, 100 mM, pH 8.0), PLP (0.1 mM), 4 °C, 120 rpm, incubation time: 3 h. Reaction conditions: $EziG^3$-AsR (22 mg, enzyme loading: 10% w w^{-1}), toluene (reaction volume: 1 mL, α_w = 0.7), **2b** (150 mM), **1a** (50 mM), 25 °C, 900 rpm, reaction time: 72 h.

entry	reaction time [h]	conversion [%][1]	ee% (*R*)
1	1	15±2	>99
2	3	34±5	>99
3	5	56±3	>99
4	8	64±3	>99
5	16	86±1	>99
6	24	91±3	>99
7	48	96±1	>99

[1] Values are depicted as actual conversion to amine product with standard deviation over three independent experiments.

4.8 Amination catalyzed by EziG-immobilized AsR-ωTA in toluene as reaction medium and applying higher substrate concentration

AsR-ωTA was immobilized (analytical scale) under standard conditions (see experimental section ─ main manuscript). The immobilized ωTA was water-equilibrated with EtOAc in the presence of hydrate salts (ca. 25 mg, ratio 1:1 w w^{-1}, Na_2HPO_3•$5H_2O$/Na_2HPO_4•$7H_2O$). The reaction was performed in toluene (α_w = 0.7, Table S11) with 50-800 mM **1a** and 1 equiv. **2b** at 25 °C (see experimental section ─ main manuscript). Conversions were determined by GC (see section 2.3).

Table S11. Transamination of **1a** catalyzed by $EziG^3$-AsR in organic solvent with higher substrate concentrations. Immobilization conditions: $EziG^3$ (Fe Amber, 20 mg, lot#EziG-130), AsR-ωTA (2 mg, 54 nmol, enzyme loading: 10% w w^{-1}), KPi buffer (1 mL, 100 mM, pH 8.0), PLP (0.1 mM), 4 °C, 120 rpm, incubation time: 3 h. Reaction conditions: $EziG^3$-AsR (22 mg, enzyme loading: 10% w w^{-1}), Na_2HPO_3•$5H_2O$/ Na_2HPO_4•$7H_2O$ (ca. 25 mg), toluene (reaction volume: 1 mL, α_w= 0.7), **2b** (concentration as specified), **1a** (concentration as specified), 25 °C, 900 rpm, reaction time: 72 h.

entry	1a [mM]	2b [mM]	conversion [%][1]	1b formed [mM]	1b formed [mg]
1	50	50	85±4	43	7
2	100	100	88±3	88	13
3	200	200	88±1	176	27
4	300	300	86±2	257	39
5	400	400	83±1	330	50
6	450	450	76±1	344	52
7	500	500	65±6	325	49
8	550	550	55±9	300	45
9	600	600	22±5	133	20
10	650	650	6±2	38	6
11	700	700	2±1	12	2
12	750	750	5±4	35	5
13	800	800	0±0	3	<1

[1] Values are depicted as actual conversion to amine product with standard deviation over three independent experiments.

4.9 Recyclability of EziG-immobilized AsR-ωTA in toluene

AsR-ωTA was immobilized (analytical scale) under standard conditions (see experimental section — main manuscript). The immobilized ωTA was water-equilibrated with EtOAc in the presence of hydrate salts (ca. 25 mg, ratio 1:1 w w^{-1}, $Na_2HPO_3{\bullet}5H_2O/Na_2HPO_4{\bullet}7H_2O$). The hydrate salt pair was added in the first reaction cycle; subsequent cycles were run with the same aliquot of hydrate salt pair unless agglomeration of the particles was observed. In that case, further addition of 10 mg of hydrate salt pair was sufficient to obtain an active catalyst. The reaction was performed in toluene (α_w = 0.7, Table S12) with 50-400 mM **1a** and 1 equiv. of **2b** at 25 °C). Conversions were determined by GC (see section 2.3). It is worth to mention that the quality of the Eppendorf reaction vessel was affected by toluene over time and it was necessary to transfer the biocatalyst to a new reaction vessel between cycle #2 and cycle #3. For that reason, no further recycling of the immobilized ωTA was attempted after the fourth reaction cycle.

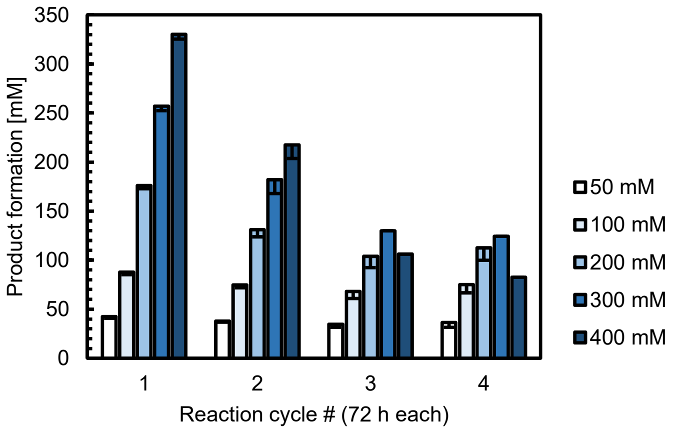

Figure S2. Recycling of $EziG^3$-AsR in toluene (α_w= 0.7) at substrate concentrations of 50 mM, 100 mM, 200 mM, 300 mM and 400 mM **1a**. Immobilization conditions: $EziG^3$ (Fe Amber, 20 mg, lot#EziG-130), AsR-ωTA (2 mg, 54 nmol, enzyme loading: 10% w w^{-1}), KPi buffer (1 mL, 100 mM, pH 8.0), PLP (0.1 mM), 4 °C, 120 rpm, incubation time: 3 h. Reaction conditions: $EziG^3$-AsR (22 mg, enzyme loading: 10% w w^{-1}), $Na_2HPO_3{\bullet}5H_2O$/ $Na_2HPO_4{\bullet}7H_2O$ (ca. 25 mg), toluene (reaction volume: 1 mL, α_w= 0.7), **2b** (concentration as specified), **1a** (concentration as specified), 25 °C, 900 rpm, reaction time: 72 h (1 reaction cycle).

Table S12. Recycling of $EziG^3$-AsR in toluene (α_w= 0.7). Immobilization conditions: $EziG^3$ (Fe Amber, 20 mg, lot#EziG-130), AsR-ωTA (2 mg, 54 nmol, enzyme loading: 10% w w^{-1}), KPi buffer (1 mL, 100 mM, pH 8.0), PLP (0.1 mM), 4 °C, 120 rpm, incubation time: 3 h. Reaction conditions: $EziG^3$-AsR (22 mg, enzyme loading: 10% w w^{-1}), $Na_2HPO_3{\bullet}5H_2O$/ $Na_2HPO_4{\bullet}7H_2O$ (ca. 25 mg), toluene (reaction volume: 1 mL, α_w= 0.7), **2b** (concentration as specified), **1a** (concentration as specified), 25 °C, 900 rpm, reaction time: 72 h (1 reaction cycle).

**entry**	**1a [mM]**	**2b [mM]**	**1b formed [mM]**				**1b formed [mg]**
			**cycle #1[1]**	**cycle #2[1]**	**cycle #3[1]**	**cycle #4[1]**	**total**
1	50	50	43±2	38±1	35±3	36±5	23
2	100	100	88±3	75±3	68±7	75±8	46
3	200	200	176±3	131±7	104±12	113±13	79
4	300	300	257±5	182±14	130±0	124±0	105
5	400	400	330±5	218±14	106±0	82±0	111

[1] Values are depicted as actual conversion to amine product with standard deviation over three independent experiments.

4.10 Continuous flow experiments

EziG[3]-AsR was applied in a continuous flow set-up (Figure 3, main manuscript) for the transamination of **1a** with **2b**. The immobilized enzyme flow reactor was prepared as depicted in the experimental section of the main manuscript. The reaction mixture was prepared in a round bottom flask (50 mL) under nitrogen atmosphere. All reaction solvents were degassed before use and water-equilibrated by stirring them with hydrate salt pairs for 1 hour at RT. The reaction solvent (50 mL) containing **1a** (20 or 50 mM) and **2b** (3 or 5 equiv.) was pumped (flow rate; 0.2 mL min^{-1}) through a pre-column containing hydrate salt pairs (4 g, Na_2HPO_3•$5H_2O$/ Na_2HPO_4•$7H_2O$) before entering the flow reactor. The outlet of the flow reactor was connected to the reactor vessel creating a closed-loop system. The flow reactor showed acceptable reproducibility for few days of operation when applying 50 mM **1a** and 3 equivalents (150 mM) **2b** in toluene (α_w = 0.7, Figure 4 – main manuscript, Table S13, entries 1-18).

To further show the reproducibility of the system, we performed the flow reaction at 20 mM **1a** and 5 equiv. **2b** (100 mM). The flow reactor was operated in cycles of 24 hours flow time as follows: the flow system was run for 24 h at a fixed flow rate (0.2 mL min^{-1}) and the conversion was determined by GC. Then, a fresh reaction mixture was prepared and the flow reaction was continued for another 24 h. The process was repeated in a continuous fashion for a total of 6 reaction cycles (Table S14, entries 1-5). The flow reactor was stored at 4 °C and used again after 4 weeks in one single cycle of 24 h. The performance was determined to be 50% of its initial activity (Table S 14, entries 6-7).

Table S13. Continuous flow experiments for the amination catalyzed by EziG[3]-AsR. Immobilization conditions: EziG[3] (Fe Amber, 400 mg, lot#EziG-130), AsR-ωTA (40 mg, 1.49 mmol, enzyme loading: 10% w w^{-1}), KPi buffer (10 mL, 100 mM, pH 8.0), PLP (0.1 mM), 4 °C, 120 rpm, incubation time: 3 h. Reaction conditions: EziG[3]-AsR (440 mg, enzyme loading: 10% w w^{-1}), $Na_2HPO_3{\bullet}5H_2O$/ $Na_2HPO_4{\bullet}7H_2O$ (ca. 600 mg), toluene (reaction volume: 50 mL, α_w = 0.7), **2b** (concentration as specified), **1a** (concentration as specified), flow rate: 0.2 mL min^{-1}, RT.

entry	Experiment no.	1a [mM]	2b [mM]	Flow time [h]	1b [%][1]	1a [%][1]
1	1	50	150	20	36	64
2				44	65	35
3				52	68	32
4				120	86	14
5	2	50	150	24	28	72
6				48	52	48
7				72	69	31
8				160	96	4
9	3	50	150	17	33	67
10				24	42	58
11				41	51	49
12				47	54	46
13			150	66	59	41
14				113	62	38
15				120	65	35
16				136	66	34
17				160	68	32
18				184	66	34

[1] Conversion calculated as described in section 3.3.

Table S13. Reproducibility experiments for the amination catalyzed by EziG[3]-AsR in flow. Immobilization conditions: EziG[3] (Fe Amber, 400 mg, lot#EziG-130), AsR-ωTA (40 mg, 1.49 mmol, enzyme loading: 10% w w^{-1}), KPi buffer (10 mL, 100 mM, pH 8.0), PLP (0.1 mM), 4 °C, 120 rpm, incubation time: 3 h. Reaction conditions: EziG[3]-AsR (440 mg, enzyme loading: 10% w w^{-1}), $Na_2HPO_3 \cdot 5H_2O$/ $Na_2HPO_4 \cdot 7H_2O$ (ca. 600 mg), toluene (α_w = 0.7), **1a** (20 mM), **2b** (100 mM), flow rate: 0.2 mL min^{-1}, RT.

entry	1a [mM]	2b [mM]	Flow time [h][1]	1b [%][2]	1a [%][2]
1	20	100	24 (24)	56	44
2	20	100	24 (48)	52	48
3	20	100	24 (72)	53	47
4	20	100	24 (96)	53	47
5	20	100	24 (120)[3]	58	42
6	20	100	24 (144)[4]	30	70
7	20	100	24 (168)	53	47

[1] total flow time of reactor is given between brackets. [2] Conversion calculated as described in section 3.3. [3] Flow reactor was stored for one day before re-use. [4] Flow reactor was stored for 28 days before re-use.

5. References

[1] P. J. Halling, *Biotechnol. Tech.* **1992**, 6, 271-276.

[2] E. Zacharis, I. C. Omar, J. Partridge, D. A. Robb, P. J. Halling, *Biotechnol. Bioeng.* **1997**, *55*, 367-374.
